# Effects of whole cigarette smoke on human beta defensins expression and secretion by oral mucosal epithelial cells

**DOI:** 10.1186/s12971-015-0029-8

**Published:** 2015-01-24

**Authors:** Wen-mei Wang, Pei Ye, Ya-jie Qian, Ya-fan Gao, Jing-jing Li, Fang-fang Sun, Wei-yun Zhang, Xiang Wang

**Affiliations:** Department of Oral Medicine, Institute and Hospital of Stomatology, Nanjing University Medical School, 30 Zhongyang Road, Nanjing, 210008 China; Department of Prosthodontics, Institute and Hospital of Stomatology, Nanjing University Medical School, Nanjing, 21008 China; Immunology and Reproduction Biology Laboratory, Medical School, Nanjing University, 22 Hankou Road, Nanjing, 210093 China

**Keywords:** Whole cigarette smoke, Human β defensin, Oral mucosa

## Abstract

**Background:**

Cigarette smoke a recognized risk factor for many systemic diseases and also oral diseases. Human beta defensins (HBDs), a group of important antimicrobial peptides expressed by the epithelium, are crucial for local defense and tissue homeostasis of oral cavity. The aim of this study was to evaluate potential effects of whole cigarette smoke (WCS) exposure on the expression and secretion of HBDs by oral mucosal epithelial cells.

**Methods:**

Immortalized human oral mucosal epithelial (Leuk-1) cells were exposed to WCS for various time periods. HBD-1, -2 and -3 expression and subcellular localization were detected by real time qPCR, immunofluorescence assay and confocal microscopy. According to the relative fluorescent intensity, the expression levels of HBD-1, -2 and -3 were evaluated by digital image analysis system. The alteration of HBD-1, -2 and -3 secretion levels was measured by the Enzyme-Linked Immunosorbent Assay.

**Results:**

WCS exposure remarkably attenuated HBD-1 expression and secretion while clearly enhanced HBD-2, -3 expression levels and HBD-2 secretion by Leuk-l cells. It appeared that there was no significant effect of WCS exposure on HBD-3 secretion.

**Conclusions:**

WCS exposure could modulate expression and secretion of HBDs by oral mucosal epithelial cells, establishing a link between cigarette smoke and abnormal levels of antimicrobial peptides. The present results may give a new perspective to investigate smoking-related local defense suppression and oral disease occurrence.

## Background

Antimicrobial peptides (AMPs) are effector molecules of the innate immune system and have antibacterial, antifungal, and antiviral effects. The human defensins, one group of small cationic AMPs, include the α-defensins of intestinal and neutrophil origin, and the β-defensins of skin, oral mucosa and other epithelia origin [1]. Human β defensins (HBDs) play important roles in innate immune and adaptive immune via their antimicrobial activity, antitumor effect, chemoattractive effect, and immunomodulation [2]. The in vitro, in vivo and human sample studies suggest that HBDs are important in the biology of the oral cavity [3]. HBD-1, -2 and -3 represent the main group of antimicrobials expressed at mucosal surfaces by epithelial cells. HBD-1 was admitted as one of the most important antibacterial defensins [4,5].

Cigarette smoke, recognized as a slow killer to human, has been considered as one of unhealthy but avoidable behaviors. All over the world, there are almost 1.3 billion active smokers, who are thought to threaten the health of nonsmokers by releasing secondhand smoke to the environment. Each year about 5–6 million people die of smoking-related diseases, such as cancers, heart diseases, stroke, lung disorders, and gastrointestinal mucosal ulcer [6]. The incidence and mortality of oral cancer are correlated strongly with two major risk factors, tobacco use and heavy alcohol use [7]. In oral cavity, cigarette smoke is also a well-recognized risk factor for periodontitis, oral candidiasis, and oral leukoplakia. The association between smoking habits and the clinical subtypes of oral lichen planus has been evaluated in a previous study [8].

Cigarette smoke is composed of approximately 5% particulate phase and 95% vapour phase by weight. In early studies, however, only the activity of the particulate phase was evaluated in routine by *in vitro* toxicological methods [9], which can not fully reflect the effects of whole cigarette smoke (WCS). Therefore, the impacts of WCS on oral epithelial cells or fibroblasts have been focused and investigated recently. Study data from Gualerzi et al. indicated that WCS had acute effects on epithelial intercellular adhesion and terminal differentiation of keratinocytes in a three-dimensional model of human oral mucosa [10]. A study of Semlali and colleagues revealed that WCS promoted human gingival epithelial cell apoptosis and inhibited cell repair processes [11]. Another study of Semlali et al. demonstrated that a single exposure to WCS produced significant morphological and functional deregulation in gingival fibroblasts [12]. The report of Colombo et al. also determined that WCS exposure resulted in oxidative damage in human gingival fibroblasts [13].

A more recent study from Pierson and colleagues confirmed that cigarette smoke extract induced differential expression levels of HBDs in human alveolar epithelial cells [14]. The study results of Semlali et al. suggested that WCS exposure up-regulated mRNA levels and protein production of HBD-2 and HBD-3 by human gingival epithelial cells [15]. As cigarette smoke enters the organism through the mouth, the anatomical cytoarchitecture of the oral mucosa is essential in providing a barrier to counteract potential harmful consequences on the whole organism [10]. HBDs are crucial for local defense and tissue homeostasis of oral cavity. However, the effects of WCS on the expression and secretion of HBDs by oral mucosal epithelial cells are still unclear. Therefore, the present study was designed to evaluate the alteration of the expression and secretion of HBDs by human oral mucosal epithelial cells exposed to WCS.

## Methods

### Cell line and cell culture

Immortalized human oral mucosal epithelial (Leuk-1) cell line was obtained from Professor Li Mao (Department of Oncology and Diagnostic Sciences, University of Maryland Dental School, Baltimore, MD, USA). Leuk-1 cells were maintained and passaged in defined keratinocyte serum-free medium (K-SFM) (GIBCO, Invitrogen, Grand Island, NY, USA) in a humidified incubator with 5% CO_2_ at 37°C. This medium was supplemented with Bovine Pituitary Extract (BPE) (25 ug/ml), epidermal growth factor (0.2 ng/ml), CaCl_2_ (0.4 mM).

### Whole cigarette smoke exposure

**Figure 1:**
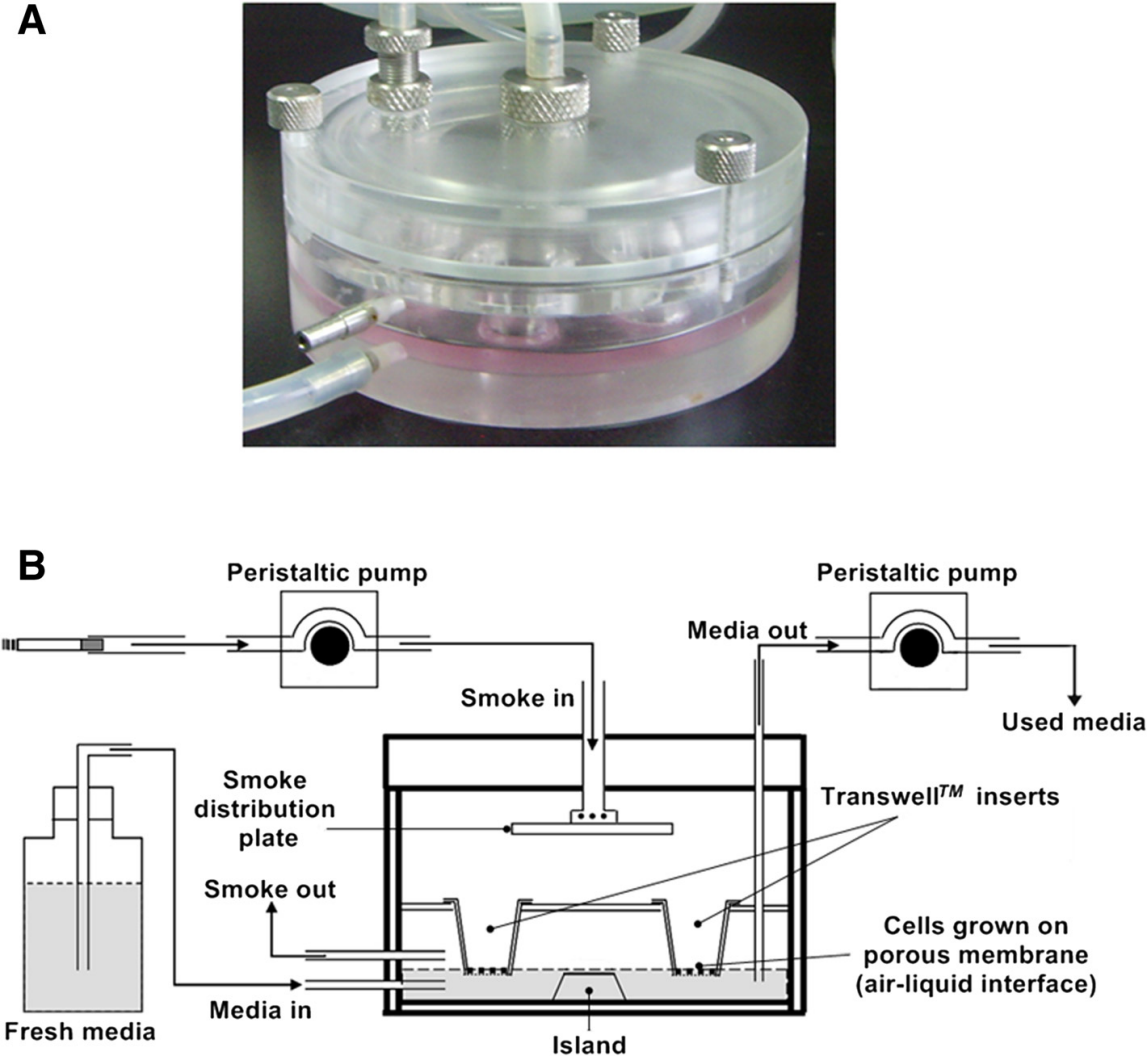
**Whole cigarette smoke (WCS) exposure apparatus. A**. WCS exposure chamber (British American Tobacco). **B**. The schematic of WCS exposure apparatus used in the present study.

First, Leuk-1 cells were seeded onto the porous membrane of Transwell™ inserts (Transwell, Corning, Schipho-Rijk, Netherlands). Cells were cultured at 37°C prior to WCS treatment. After 24 h, the Transwell™ inserts were taken out and transferred into a WCS exposure chamber (Figure 1A). As previously described [9,16-20], the WCS exposure chamber (UK patent publication WO 03/100417/ A1, British American Tobacco, UK) was used to expose cell cultures to mainstream cigarette smoke. The temperature inside the chamber remained stable. The chamber can accommodate 6 × 12 mm inserts, which allow cells to grow on the porous membrane at the air-liquid interface. The cells were cultured on Transwell™ inserts which were housed in the exposure chamber and were exposed apically to evenly distributed cigarette smoke. Kentucky 3R4F research-reference filtered cigarettes (The Tobacco Research Institute, University of Kentucky, Lexington, KY), one of which contains 0.73 mg of nicotine and 9.4 mg of tar, were used in the present study. A peristaltic pump (LongerPumper, USA) was used to control the speed of smoke import at 20 rpm. Cigarette smoke was exhausted in the chamber and old smoke was replaced by fresh smoke from the next puff. A peristaltic pump was adopted to regulate the contact relationship between the media and porous membrane, which prevented flooding of the cell monolayer and disruption of the air-liquid interface. Fresh media were introduced basally and used media were drained away from the chamber by a peristaltic pump at 80 rpm. Media flow is unidirectional to avoid build up of smoke toxicants, which may affect exposure conditions basally. Following WCS exposure, the Transwell™ inserts were transferred to a 12-well plate as before at 37°C in 5% CO_2_ for 6 h. Figure 1B showed a schematic of the *in vitro* WCS exposure apparatus.

In the experimental group, cell cultures were exposed to WCS for 5 min, 10 min, 20 min, and 40 min, respectively. Based on our preliminary study, the results confirmed that there was no significant difference in expression and secretion levels of HBD-1, -2 and -3 of Leuk-1 cells exposed to filtered air for 5 min, 10 min, 20 min, and 40 min. Thus, cell cultures exposed to filtered air for 10 min were set as the control group.

### Real time quantitative reverse transcription PCR (qRT-PCR)

Total cellular RNA was extracted by using TRIzol reagent (Invitrogen, Carlsbad, CA, USA) and 2 μg of RNA was used to synthesize the first-strand cDNA in 20 μl of reaction volume using RevertAid First Strand cDNA Synthesis Kit (Thermo, Grand Island, NY, USA) according to the manufacturer’s protocols. Real-time PCR analyses ware performed using an ABI 7300 Real Time PCR System (Applied Biosystem, Foster City, CA), and PCR amplifications were performed using the SYBR Green PCR Master Mix (Roche, Mannheim, Germany) according to the manufacturer’s instruction. Amplification conditions were as follows: 50°C for 2 min, 95°C for 10 min, 40 cycles of 95°C for 15 s, 58°C for 30 s, and 72°C for 30 s, followed by melting curve analysis, by which the specificity of primers was confirmed. The data are expressed as relative mRNA levels and were normalized to GAPDH. Fold changes in expression of each gene were calculated by a comparative threshold cycle (Ct) method using the formula 2^−(ΔΔCt)^. The experiment was repeated three times. The primers used for the PCR amplifications are listed as follows: HBD-1 forward TCA TTA CAA TTG CGT CAG CAG, reverse TTG CAG CAC TTG GCC TTC [21]; HBD-2 forward TCC TCT TCT CGT TCC TCT TCA, reverse AGG GCA AAA GAC TGG ATG AC [21]; HBD-3 forward CCA TTA TCT TCT GTT TGC TTT GCT C, reverse CCG CCT CTG ACT CTG CAA TAA TA [22]; GAPDH forward GCA CCG TCA AGG CTG AGA AC, reverse TGG TGA AGA CGC CAG TGG A [23].

### Immunofluorescence and confocal microscopy

To evaluate intracellular expression levels of HBD-1, -2 and -3 by using immunofluorescence and confocal microscopy, Leuk-1 cells were cultured on glass coverslips for 24 h. Then each coverslip was transferred onto the bottom of Transwell^TM^ insert, respectively. Subsequently, Leuk-1 cells on coverslips were exposed with WCS or filtered air (control) in above-mentioned exposure chamber. Following treatment, Leuk-1 cells on coverslips were recovered in fresh K-SFM at 37°C for 6 h. Next, Leuk-1 cells on coverslips were washed with PBS and fixed in 4% paraformaldehyde for 15 min at room temperature. After being washed in PBS, the cells were permeabilized in 0.5% (v/v) Triton X-100 in PBS, washed, and blocked with 5% BSA in PBS-0.1% Tween 20 for 1 h at 37°C. Next, the cells were exposed overnight at 4°C to primary antibodies. Primary antibodies against the following proteins were used: mouse anti-HBD-1 antibody (1:100), and rabbit anti- HBD-2 antibody (1:100) were purchased from Abcam (Cambridge, UK); rabbit anti-HBD-3 antibody (1:100) was purchased from Novus (Littleton, CO, USA). The next day, coverslips were washed with PBS and then incubated with Dylight 488 (green)-labeled goat-anti-mouse secondary antibody or Alexa Fluor 555 (red)-labeled goat-anti-rabbit secondary antibody for 1 h at room temperature. To stain the nuclei, 1 μg/ml (w/v) 4′, 6-diamidino-2-phenylindole (DAPI, Sigma, USA) was added for 5 min, and slides were examined by a confocal laser scanning microscope (FluoView FV10i, Olympus, Japan).

### Digital image analysis

Digital image analysis of immunostaining results was performed as described previously with minor modification [24]. Altered intracellular levels of HBD-1, -2 and -3 were evaluated by fluorimetry in Leuk-1 cells. Fluorescence imaging experiments were performed at room temperature and images were acquired with confocal laser scanning microscope (FluoView FV10i, Olympus, Japan), with a 600 × objective. Images were analyzed with the Image J software. Values represent the average of the mean fluorescence intensity measured in randomly distributed fields (each field represented a region of interest, ROI) selected on independent coverslips (10 ROIs for each coverslip). All ROIs were of the same size in the experiment. ROIs always included multiple cells and only adherent cells were considered in the analysis. In the current study, the relative fluorescence intensity was used to represent expression levels of HBD-1, -2 and -3. The relative fluorescence intensity in control group was considered as “1.0”. The ratio of the mean fluorescence intensity of each exposure group to that of control group was regarded as the relative fluorescence intensity of corresponding exposure group.

### Subcellular localization of HBD-1, -2 and -3

Leuk-1 cells grown on coverslips were exposed with WCS or filtered air (control) in Transwell™ inserts. Following treatment, Leuk-1 cells on coverslips were recovered in fresh K-SFM at 37°C for 6 h. Immunofluorescence staining was performed as above-mentioned methods. Subcellular localization of HBD-1, -2 and -3 in Leuk-1 cells was detected using confocal microscopy.

### Enzyme-linked immunosorbent assay (ELISA)

To further study the effects of WCS exposure on HBDs releases from human oral epithelial cells, ELISA assays were performed to detect HBDs levels in the supernatant of Leuk-1 cells culture following WCS exposure. First, Leuk-1 cells were seeded onto the porous membrane of Transwell™ inserts at a density of 3 × 10^5^ cells/well in 0.5 ml of K-SFM medium, which were inserted into a 12-well plate. Then K-SFM was added into the underlayer wells for 1.5 ml per well. Cells were cultured at 37°C for prior to WCS treatment. After 24 h, the inserts were taken out and transferred into the WCS exposure chamber. Following WCS exposure, the inserts were transferred into a 12-well plate as before at 37°C in 5% CO_2_ for 6 h.

To detect the amount of HBD-1, -2 and -3 secreted by Leuk-1 cells, ELISA kits were used to measure the levels of HBD-1, -2 and -3 in cell culture supernatant. The HBD-1, -2 and -3 ELISA Kits were purchased from Jingtian (Shanghai, China). HBD-1, -2 and -3 standard substances for controls (3200 pg/ml) were used to construct standard curves (Jingtian, Shanghai, China). HBD-1, -2 and -3 levels in leuk1 cell supernatant were measured according to the manufacturer’s protocols. Absorbances were read at 450 nm and 570 nm using a microplate reader, the absorbance at 570 nm was subtracted from the absorbance at 450 nm.

### Statistical analysis

Statistical analysis was performed using SPSS 15.0. Data are presented as mean ± SE. Significance was analyzed using the Student’s *t*-test or One-Way ANVOA test. *P* value less than 0.05 was considered significant.

## Results

### WCS exposure down-regulated mRNA level of HBD-1 and up-regulated mRNA levels of HBD-2 and -3 in Leuk-1 cells

**Figure 2:**
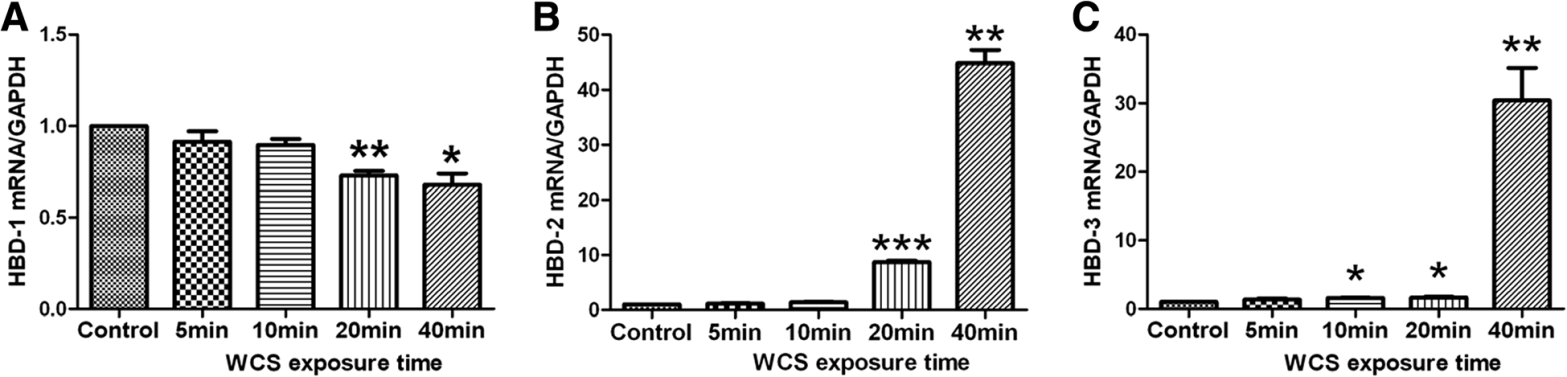
**WCS exposure down-regulated HBD-1 mRNA level and up-regulated HBD-2, −3 mRNA levels in Leuk-1 cells.** HBD-1, −2 and −3 mRNA levels were assayed by qRT-PCR. **A.** HBD-1 mRNA level was markedly down-regulated in Leuk-1 cells following 20 min- and 40 min-WCS exposure compared with control. **B.** HBD-2 mRNA level was dramatically up-regulated in Leuk-1 cells following 20 min- and 40 min-WCS exposure compared with control. **C.** HBD-3 mRNA level was remarkably up-regulated in Leuk-1 cells following 10 min-, 20 min- and 40 min-WCS exposure compared with control. The qPCR data were expressed as means ± SE (n = 3). Statistical significance: *P < 0.05, vs. control; **P < 0.01, vs. control; ***P < 0.001, vs. control.

The qRT-PCR results indicated that HBD-1 mRNA level was markedly down-regulated in Leuk-1 cells following 20 min- and 40 min-WCS exposure compared with control (*P* < 0.01 and *P* < 0.05, respectively) (Figure 2A). Our present data also suggested that HBD-2 mRNA level was dramatically up-regulated in Leuk-1 cells following 20 min- and 40 min-WCS exposure compared with control (*P* < 0.001 and *P* < 0.01, respectively) (Figure 2B). HBD-3 mRNA level was remarkably up-regulated in Leuk-1 cells following 10 min-, 20 min- and 40 min-WCS exposure compared with control (*P* < 0.05, *P* < 0.05, and *P* < 0.01, respectively) (Figure 2C).

### WCS exposure reduced protein level of HBD-1 in Leuk-1 cells

**Figure 3:**
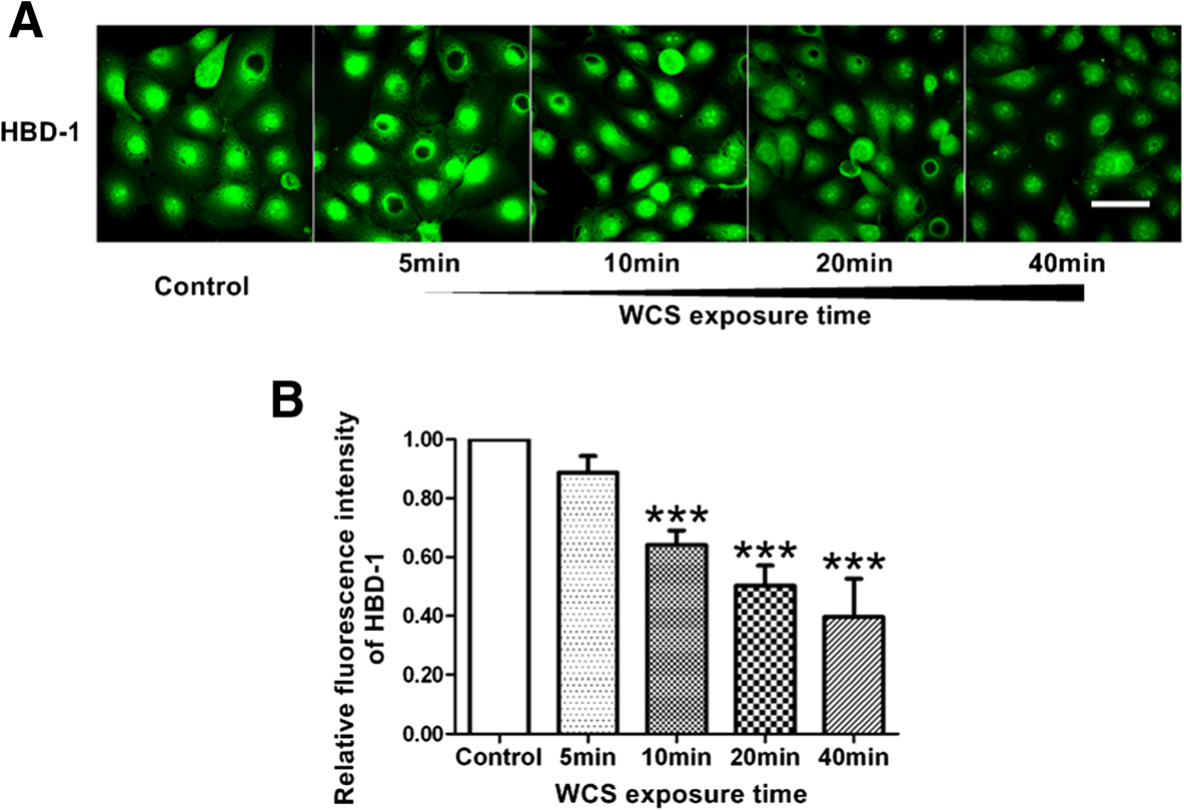
**WCS exposure reduced HBD-1 level in Leuk-1 cells. A**. Representative immunofluorescence stainings of HBD-1 (green) in Leuk-1 cells in control group and WCS exposure groups. Scale bar = 50 μm. **B**. The relative fluorescence intensity was used to represent protein level of HBD-1. HBD-1 level significantly decreased in Leuk-1 cells following 10 min-, 20 min- and 40 min-WCS exposure compared with control. Statistical significance: ***P < 0.001, vs. control.

As these proteins of HBD-1, -2 and -3 have extremely small size (about 4 ~ 7 kD), the immunoblotting of HBDs remains a challenge. Thus, the immunofluorescence assay and digital image analysis were preformed in the present study to access the protein levels of HBD-1, -2 and -3. As shown in Figure 3, HBD-1 level significantly decreased in Leuk-1 cells following 10 min-, 20 min-, or 40 min-WCS exposure compared with control (*P* < 0.001, *P* < 0.001, and *P* < 0.001, respectively).

### WCS exposure increased protein levels of HBD-2 and HBD-3 in Leuk-1 cells

**Figure 4:**
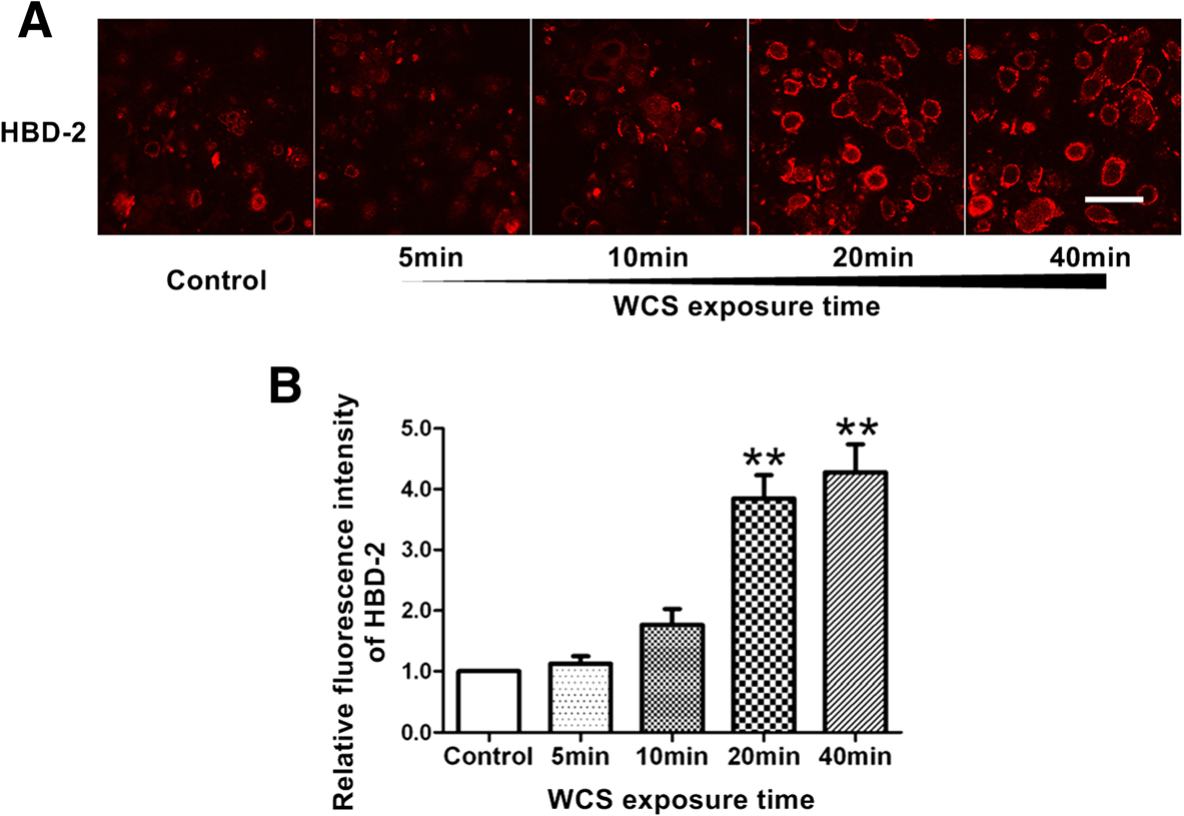
**WCS exposure increased HBD-2 level in Leuk-1 cells. A**. Representative immunofluorescence stainings of HBD-2 (red) in Leuk-1 cells in control group and WCS exposure groups. Scale bar = 50 μm. **B**. The relative fluorescence intensity was used to represent protein level of HBD-2. HBD-2 level remarkably increased in Leuk-1 cells following 20 min- and 40 min-WCS exposure compared with control. Statistical significance: **P < 0.01, vs. control.

**Figure 5:**
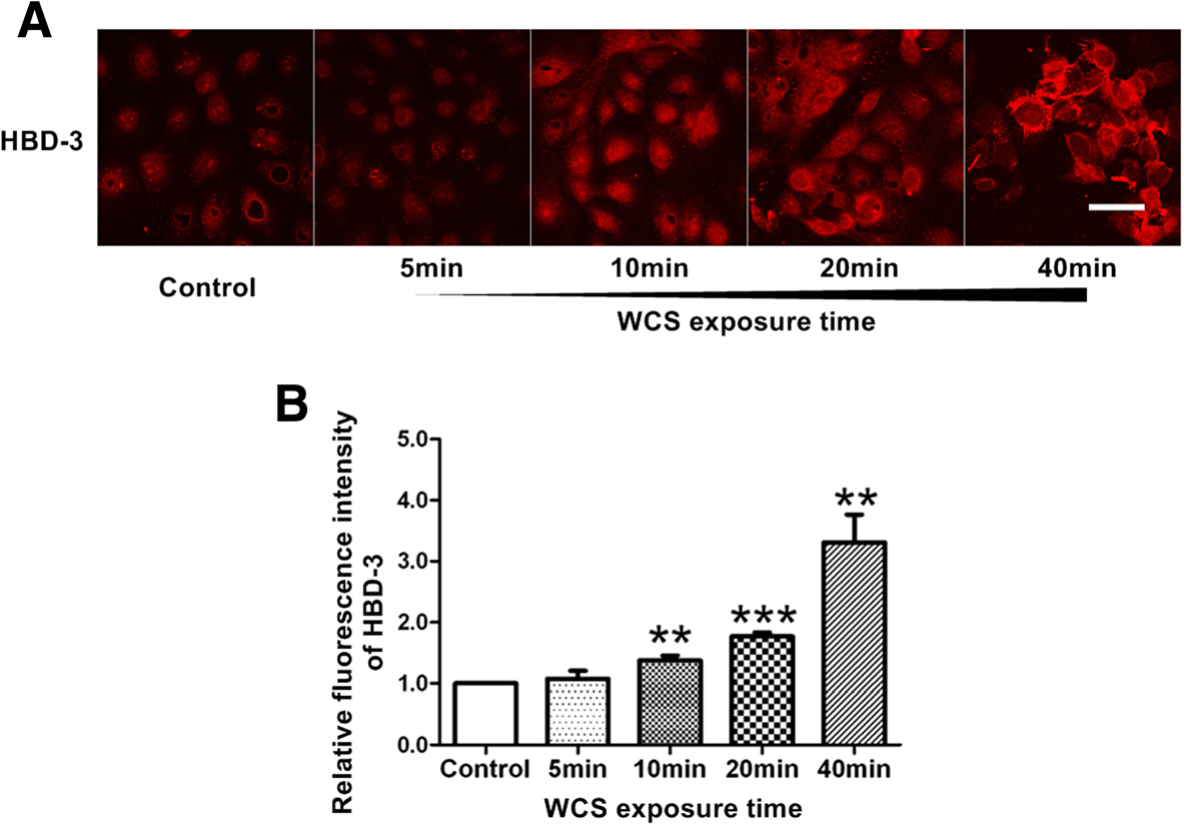
**WCS exposure augmented HBD-3 level in Leuk-1 cells. A**. Representative immunofluorescence stainings of HBD-3 (red) in Leuk-1 cells in control group and WCS exposure groups. Scale bar = 50 μm. **B**. The relative fluorescence intensity was used to represent protein level of HBD-3. HBD-3 level was markedly augmented in Leuk-1 cells following 10 min-, 20 min- and 40 min-WCS exposure compared with control. Statistical significance: **P < 0.01, vs. control; ***P < 0.001, vs. control.

In the present study, our results indicated that HBD-2 level remarkably increased in Leuk-1 cells following 20 min- and 40 min-WCS exposure compared with control (*P* < 0.01 and *P* < 0.01, respectively) (Figure 4). Our data also suggested that HBD-3 level markedly augmented in Leuk-1 cells following 10 min-, 20 min- and 40 min-WCS exposure compared with control (*P* < 0.01, *P* < 0.001, and *P* < 0.01, respectively) (Figure 5).

### Subcellular localization of HBD-1, -2, and -3

**Figure 6:**
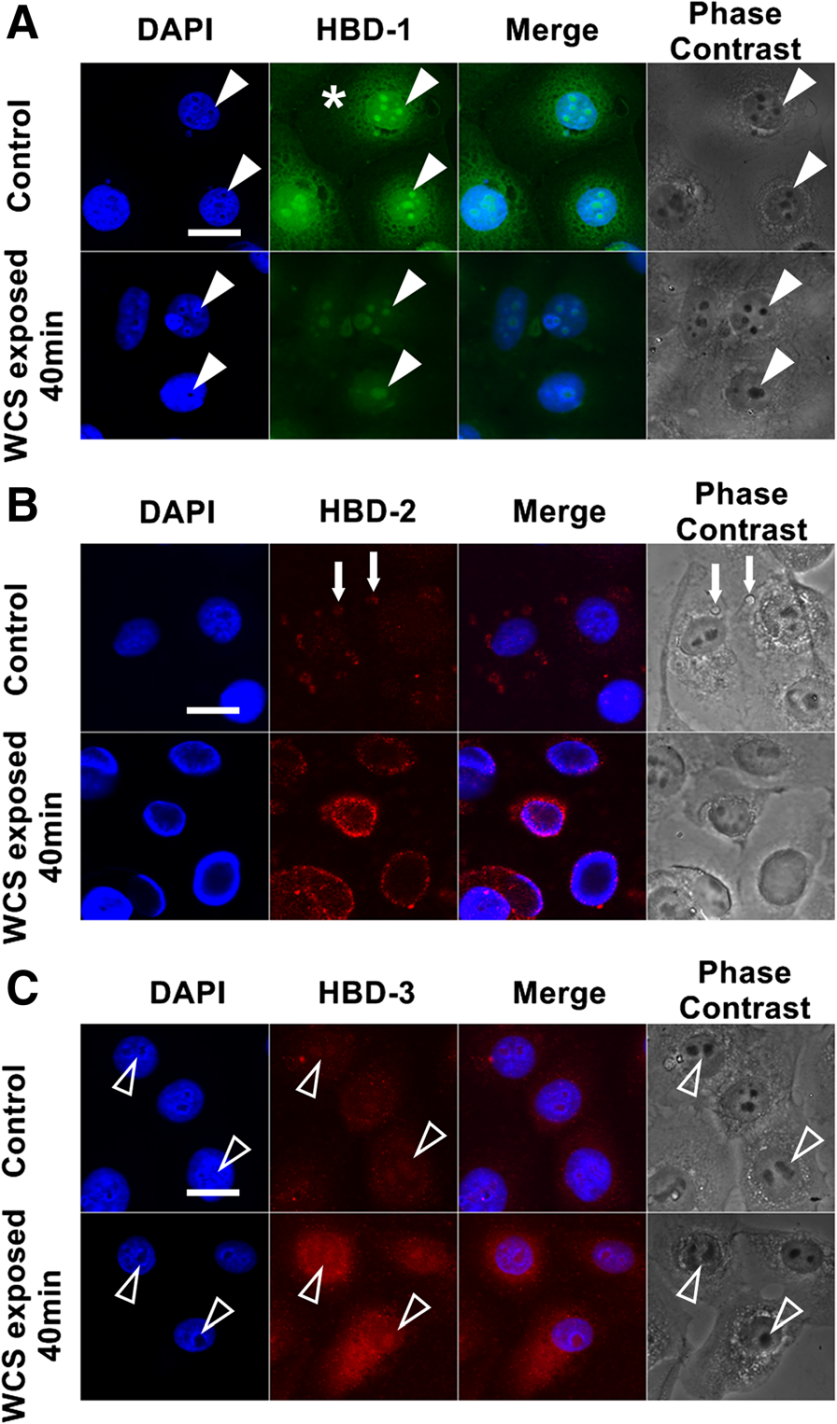
**Subcellular localization of HBD-1, -2 and -3 in Leuk-1 cells. A**. HBD-1 (green) was mainly localized in perinuclear cytoplasm and nuclei. Reticular immunostaining of HBD-1 was clearly observed in perinuclear cytoplasm (asterisk) and strong expression of HBD-1 was especially noted in nucleoli area (closed arrowhead). Following 40 min-WCS exposure, reticular immunostaining of HBD-1 in perinuclear cytoplasm nearly disappeared and the expression of HBD-1 in nucleoli area was weak (closed arrowhead). **B**. The weak expression of HBD-2 (red) was mainly in perinuclear cytoplasm. Some expression of HBD-2 could also be detected in cytoplasmic vesicles (arrow). Following 40 min-WCS exposure, the expression of HBD-2 dramatically enhanced and localized in perinuclear cytoplasm. **C**. HBD-3 (red) was mainly localized in perinuclear cytoplasm and nucleoli area (open arrowhead), which was expressed weakly. Following 40 min-WCS exposure, the expression of HBD-3 dramatically augmented and still localized in perinuclear cytoplasm and nucleoli area (open arrowhead). Scale bar = 20 μm.

Through immunofluorescence and confocal microscopy, the subcellular localization of HBD-1, -2, and -3 in Leuk-1 cells was determined in the present study. The subcellular localization of HBD-1 was mainly in perinuclear cytoplasm and nuclei. Reticular immunostaining of HBD-1 was clearly observed in perinuclear cytoplasm and strong expression of HBD-1 was especially noted in nucleoli area. Following 40 min-WCS exposure, however, the expression of HBD-1 in perinuclear cytoplasm almost disappeared and the expression of HBD-1 in nucleoli area was weak (Figure 6A).

The weak expression of HBD-2 was observed mainly in perinuclear cytoplasm. Some expression of HBD-2 could also be detected in cytoplasmic vesicles. Following 40 min-WCS exposure, the expression of HBD-2 localized in perinuclear cytoplasm dramatically enhanced and (Figure 6B). The subcellular localization of HBD-3 was mainly in perinuclear cytoplasm and nucleoli area, which was expressed weakly. Following 40 min-WCS exposure, the expression of HBD-3 dramatically augmented and still localized in perinuclear cytoplasm and nucleoli area (Figure 6C).

### WCS exposure attenuated HBD-1 secretion by Leuk-l cells

**Figure 7:**
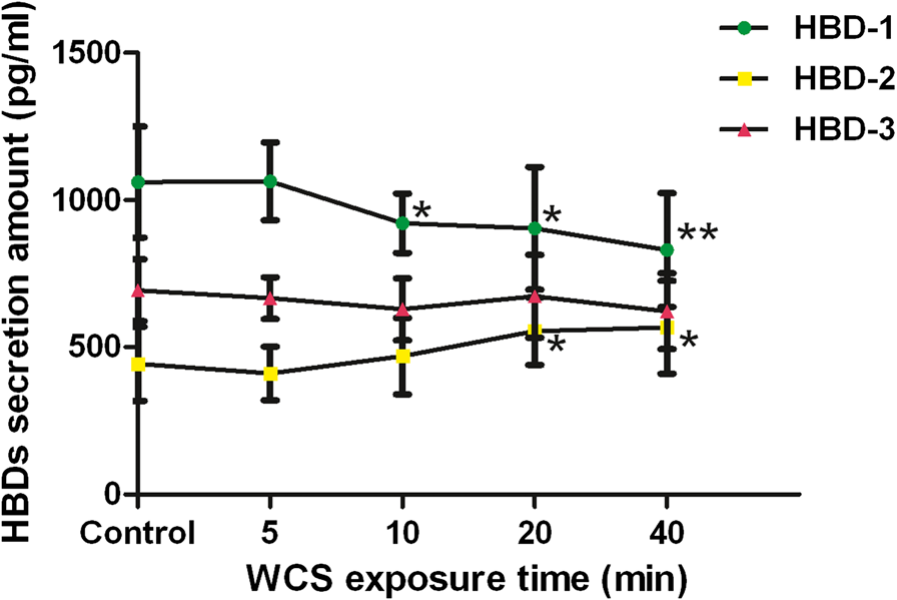
**WCS exposure attenuated HBD-1 secretion and enhanced HBD-2 secretion by Leuk-l cells.** Compared with the basal secretion level in control group, 10 min-, 20 min- and 40 min-WCS exposure induced significant down-regulation of HBD-1 secretion by Leuk-1 cells. Compared with the basal secretion level in control group, 20 min- and 40 min-WCS exposure significantly enhanced HBD-2 secretion by Leuk-1 cells. There was no significant difference of HBD-3 secretion between WCS exposure groups and control group. ELISA data were expressed as means ± SE (n = 12/group). Statistical significance: *P < 0.05, vs. control; **P < 0.01, vs. control.

As shown in Figure 7, compared with the basal secretion in control group, 10 min- and 20 min-WCS exposure induced clear down-regulation of HBD-1 secretion by Leuk-1 cells (*P* < 0.05 and *P* < 0.05, respectively). Moreover 40 min-WCS exposure dramatically attenuated HBD-1 secretion by Leuk-1 cells compared with the control group (*P* < 0.01).

### Alteration of the secretion of HBD-2 and HBD-3 by Leuk-1 after WCS exposure

As shown in Figure 7, compared the basal secretion in control group, 20 min- and 40 min-WCS exposure significantly enhanced HBD-2 secretion by Leuk-1 cells (*P* < 0.05 and *P* < 0.05, respectively). No significant difference of HBD-3 secretion was found between WCS exposure groups and control group.

## Discussion

Mainstream cigarette smoke is an aerosol consisting of a gas/vapor and particulate phase which contains over 4,000 individual chemical constituents [25]. Traditionally, two main methods of cigarette smoke exposure are used for *in vitro* studies. One method is to collect the particulate phase with a Cambridge filter pad, which is referred to as total particulate matter (TPM) or cigarette smoke condensate (CSC). The other method is to isolate water-soluble cigarette smoke components from both particulate and gas phases by bubbling cigarette smoke through PBS or cell culture medium, which is referred to as aqueous cigarette smoke extract (CSE). However, both two methods of collecting smoke phases have limitations. Capturing particulate fraction neglects the gas phase components of the mixture, while bubbling of cigarette mainstream smoke through PBS or medium fails to capture a significant amount of the particulate phase [26].

To better understand the toxicological and biological effects of cigarette smoke, the exposure system of WCS which consists of particulate and gas/vapor phase, has been developed in the past decade. A novel WCS exposure system has been established and designed to expose human epithelial cells cultured on Transwell™ inserts to mainstream WCS (UK patent publication WO 03/100417/ A1) [9,16-20]. This system allows all phases of cigarette smoke including particulate and gas to be assessed in combination.

All the cells within oral cavity are the first to be exposed to cigarette smoke and oral cavity may be a potential approach for the spread of toxins to other organs of the body [27]. Upon entrance into the oral cavity, cigarette smoke reaches the oral mucosa where epithelial cells act as the first line of defense and play a crucial role in maintaining host homeostasis. Alike to other microbial barriers, oral mucosal epithelial cells play a critical role in host defense and innate immune. Previously published results demonstrated the role of HBDs in maintaining homeostatic levels of commensal bacteria, in protecting against colonization of pathogenic microbes, both under constitutive circumstances, and in response to the interaction of the pathogen with the epithelial cell [3]. These strongly supported the crucially defensive role of HBDs in the oral cavity. Although 28 HBDs have been found till now, the expression and effects of HBD-1, -2 and -3 have been most investigated [28].

In the present study, our results revealed for the first time that WCS exposure attenuated HBD-1 expression and secretion by human oral mucosal epithelial cells. Wolgin et al. found that the mRNA level of HBD-1 was significantly reduced in gingival samples from smokers compared to that from non-smokers [21]. Shibata et al. determined that the mRNA level of mouse β defensin (mBD)-1, a functional homolog of HBD-1, decreased in the lungs of cigarette smoke-exposed mice compared with that of air-exposed mice [29]. As one of the most important AMP in epithelial cells, HBD-1 is thought to be constitutively expressed and persistently secreted by various epithelial cells of many organs [4,30]. It has been well demonstrated that HBD-1 played crucial roles in antitumor, antimicrobial activity, homeostasis maintenance, wound healing, and immunomodulatory effect. Based on existed evidences, the present results may give a new clue to understand smoking-related local defense suppression and oral diseases occurrence.

In the present study, our results showed that WCS exposure enhanced the expression of HBD-2 and HBD-3 and augmented HBD-2 secretion by human oral mucosal epithelial cells. The data of Semlali et al. suggested that WCS exposure for 15–30 min increased mRNA levels and protein production of HBD-2 and HBD-3 by human gingival epithelial cells [15]. The results of Shibata et al. indicated that mBD-2 and mBD-3 expression increased in the lungs of cigarette smoke-exposed mice compared with air-exposed mice [29]. The study results of Chen et al. indicated that cigarette smoke enhanced rat β defensin (rBD)-2 expression in rat airways via NF-κB activation [31]. Normally, only a small amount HBD-2 and HBD-3 express in epithelial cells. HBD-2 and HBD-3 could be induced by pathogenic bacteria and proinflammatory cytokines, such as TNF-α, IL-1β, IFN-γ [28,32]. Our current results are consistent with the previous data in the literature and supported that WCS exposure could up-regulate HBD-2 and HBD-3 expression. Semlali et al. found that WCS exposure promoted HBD-2, HBD-3, IL-1β, and IL-6 expression through the ERK1/2 and NF-κB pathways. The increased expression of HBD-2 and HBD-3 following WCS exposure might be due to proinflammatory response of cells [15]. Cigarette smoke may directly stimulate novel HBDs production, leading to enhancement of IL-1β, which in turn could further stimulate additional HBDs production, in a paracrine or autocrine loop [14]. Stimulation of oral squamous cell carcinoma (BHY-OSCC) cell line with HBD-1 resulted in reduction of cell proliferation, whereas HBD-2 and HBD-3 stimulation caused promotion of cell proliferation, indicating that HBD-1 might function as a tumor suppressor gene, while hBD-2 and -3 might be protooncogenes in OSCC [33]. Exposure to cigarette smoke is thought to be harmful to host respiratory defenses through multiple modes of action [34]. Our present results revealed that WCS exposure had differential effects on expression and secretion of HBDs by oral mucosal epithelial cells, suggesting cigarette smoke-induced abnormal expression of HBDs is involved in some oral diseases and should be further investigated mechanistically.

Some present data on HBD-2 and HBD-3 were not in agreement with some of previous reports. Wolgin et al. found that the mRNA level of HBD-2 was significantly reduced in gingival samples of smokers compared to that of non-smokers [21]. Mahanonda et al. reported that CSE markedly reduced *Porphyromonas gingivalis* lipopolysaccharide-stimulated HBD-2 expression in human gingival epithelial cells *in vitro* [35]. The study results of Semlali et al. suggested that WCS exposure significantly augmented the secretion of HBD-3 by human gingival epithelial cells. These conflicting findings between the literature and the present study may be partly explained by the difference between human tissue specimen and cell culture. Generally, influencing factors in *in vitro* study are much fewer than that in *in vivo* study, which include systemic factors, immunomodulation, microorganisms, quantitation of cigarette smoke treatment, etc. Furthermore, the experimental model and cell type used in each study may be of considerable importance [15]. CSE and WCS exposure may induce different responses of cells. Similarly, different study apparatuses or methods of WCS exposure may also lead to potentially conflicting results.

In the present results, the difference of altered extent of HBDs levels was observed between the expression level in Leuk-1 cells and the secretory level in supernatant of cell culture. The altered extent of HBDs at secretory level was more minor than that at expression level. WCS exposure remarkably enhanced the expression HBD-3, while the secretion of HBD-3 seemed not to be significantly impacted. Mainly, the difference could result from the regulation of antimicrobial peptide expression at transcriptional, post-transcriptional and post-translational levels [36,37]. Additionally, this difference may be explained that only a part of intracellular HBDs could be released into supernatant of cell culture. In the post-translational translocation process, the synthesis and translocation of the preproteins are uncoupled [38]. By potentially modulating the production and release of HBDs and other antimicrobial peptides, oral mucosal epithelial cells might construct and control the defensive response system to microbe.

## Conclusion

The present study revealed that WCS exposure could modulate HBDs expression and secretion by oral mucosal epithelial cells, which might be a link between cigarette smoke and oral diseases. Establishing a relationship between smoking and abnormal levels of antimicrobial peptides is crucial to understand local defense dysfunction in cigarette smokers, which might provide a new perspective to interpret the pathology of tobacco-induced diseases.

## Abbreviations

WCS: Whole cigarette smoke
HBD: Human β defensin
ELISA: Enzyme-linked immunosorbent assay
AMP: Antimicrobial peptide
TPM: Total particulate matter
CSC: Cigarette smoke condensate
CSE: Cigarette smoke extract
